# Quadricuspid Aortic Valve: Three Cases Report and Literature Review

**DOI:** 10.21470/1678-9741-2018-0125

**Published:** 2019

**Authors:** Elinthon Tavares Veronese, Carlos Manuel de Almeida Brandão, Samuel Padovani Steffen, Pablo Pomerantzeff, Fabio B. Jatene

**Affiliations:** 1 Cardiovascular Surgery Division, Instituto do Coração do Hospital das Clínicas da Faculdade de Medicina da Universidade de São Paulo (InCor-HCFMUSP), São Paulo, SP, Brazil.

**Keywords:** Congenital Heart Defects, Aortic Valve - Pathology, Aortic Valve - Abnormalities

## Abstract

Quadricuspid aortic valve (QAV) is a rare cardiac malformation. Many cases are incidentally diagnosed in aortic surgeries or autopsies and it usually appears as an isolated anomaly. The most widely classification used is the one by Hurwitz and Roberts^[1]^, which divides 7 alphabetical subtypes based on the cusps size. The aim of this report is to describe three different anatomic presentations of this rare aortic valve anomaly.

**Table:** 

Abbreviations, acronyms & symbols	
CHD	= Coronary Heart Desease
LVEF	= Left ventricular ejection fraction
QAV	= Quadricuspid aortic valve

## INTRODUCTION

Quadricuspid aortic valve (QAV) is a rare cardiac malformation, with an incidence of 0.003 to 0.043% of all congenital heart defects^[1,2]^. Many cases are incidentally diagnosed in aortic surgeries or autopsies and it usually appears as an isolated anomaly. The diagnosis is usually late, on the dependence of the signs and symptoms related to the aortic valve dysfunction. The surgical treatment is usually indicated in the fifth or sixtieth decades and follow the current guidelines for valvular diseases.

## CASES REPORT

### Case 01

**Fig. 1:**
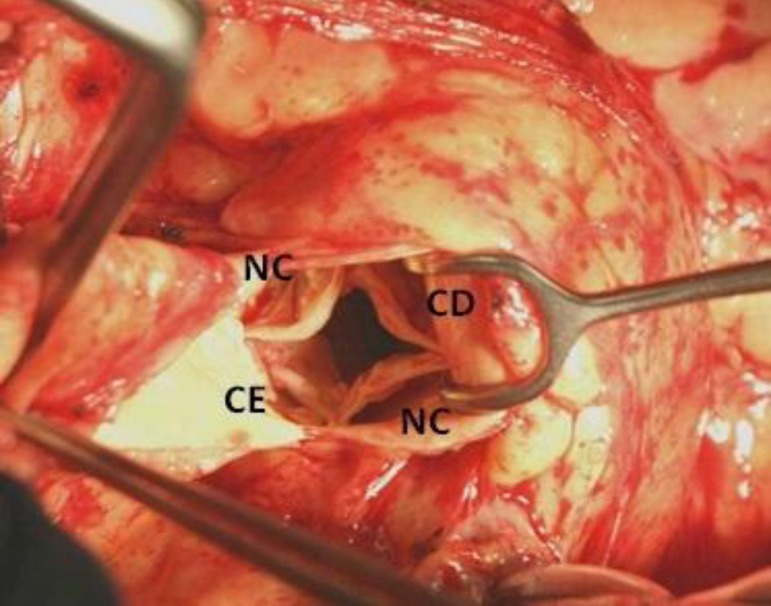
Intraoperative view of Type-C Hurwitz and Roberts QAV. CE=left coronary cusp; CD=right coronary cusp; NC=non coronary cusp.

A 53 year-old male patient with a progressive worsening of symptoms of dyspnea in the last three years was admitted to our institution for a routine evaluation. At the time, he had a marked limitation of physical activity associated to orthopnea, paroxysmal nocturnal dyspnea and an isolated episode of syncope. The physical examination demonstrated both systolic and diastolic murmurs in the right upper sternal border with radiation to both carotid arteries. Transthoracic echocardiogram revealed a severe stenotic and mild/moderate insufficient aortic valve with left ventricular ejection fraction (LVEF) preserved (54%). The intraoperative transesophageal echocardiogram revealed a quadricuspid aortic valve. In the surgical field, the aortic valve was composed by four retracted and calcified cusps and it was classified as a Type C in the Hurwitz and Roberts classification. There were two equal larger cusps and two equal smaller cusps (Figure 1). Due to the calcification and retraction of all leaflets, the patient was submitted to a convencional aortic valve replacement.A Braile^®^ 25mm Bovine Pericardium Bioprosthesis was implanted. The patient had a short in-hospital postoperative recovery and remains asymptomatic after five years of follow-up.

### Case 2

**Fig. 2:**
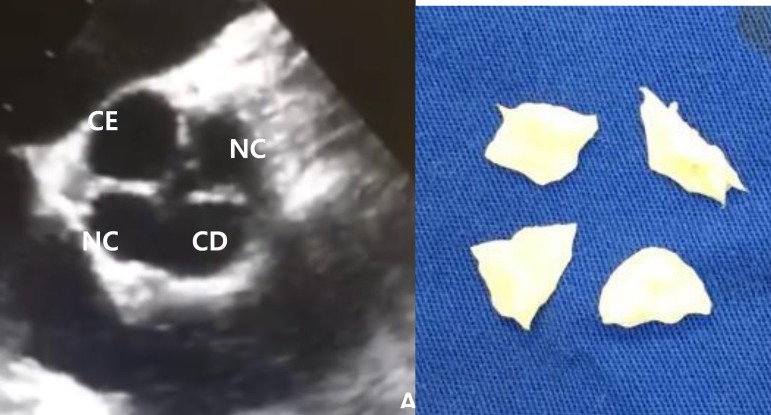
(A)Transesophageal echocardiogram showing of Type-E Hurwitz and Roberts QAV. (B)Image of the four resected cusps. CE=left coronary cusp; CD=right coronary cusp; NC=non coronary cusp.

A 57 year-old female patient with a progressive worsening of dyspnea in the previous six months was admitted to our institution for an elective surgery. At the time she had a marked limitation of physical activity. The physical examination demonstrated a diastolic murmur +3/+6 at the left middle sternal border. A Transthoracic Echocardiogram revealed a severe insufficient aortic valve with LVEF preserved (57%); however the exam did not describe any malformation in the aortic valve. The intraoperative transesophageal echocardiogram revealed a quadricuspid aortic valve (Figure 2A). In the surgical field, the aortic valve was composed by four retracted cusps (Figure 2B) and it was classified as a Type E in the Hurwitz and Roberts classification. There were three equal cusps and one larger cusp. Due to the cusps retraction, the patient was submitted to a conventional aortic valve replacement. A Braile^®^ 21mm Bovine Pericardium Bioprosthesis was implanted. The patient had a short in-hospital postoperative recovery and remains asymptomatic after six months of follow-up.

### Case 3

**Fig. 3:**
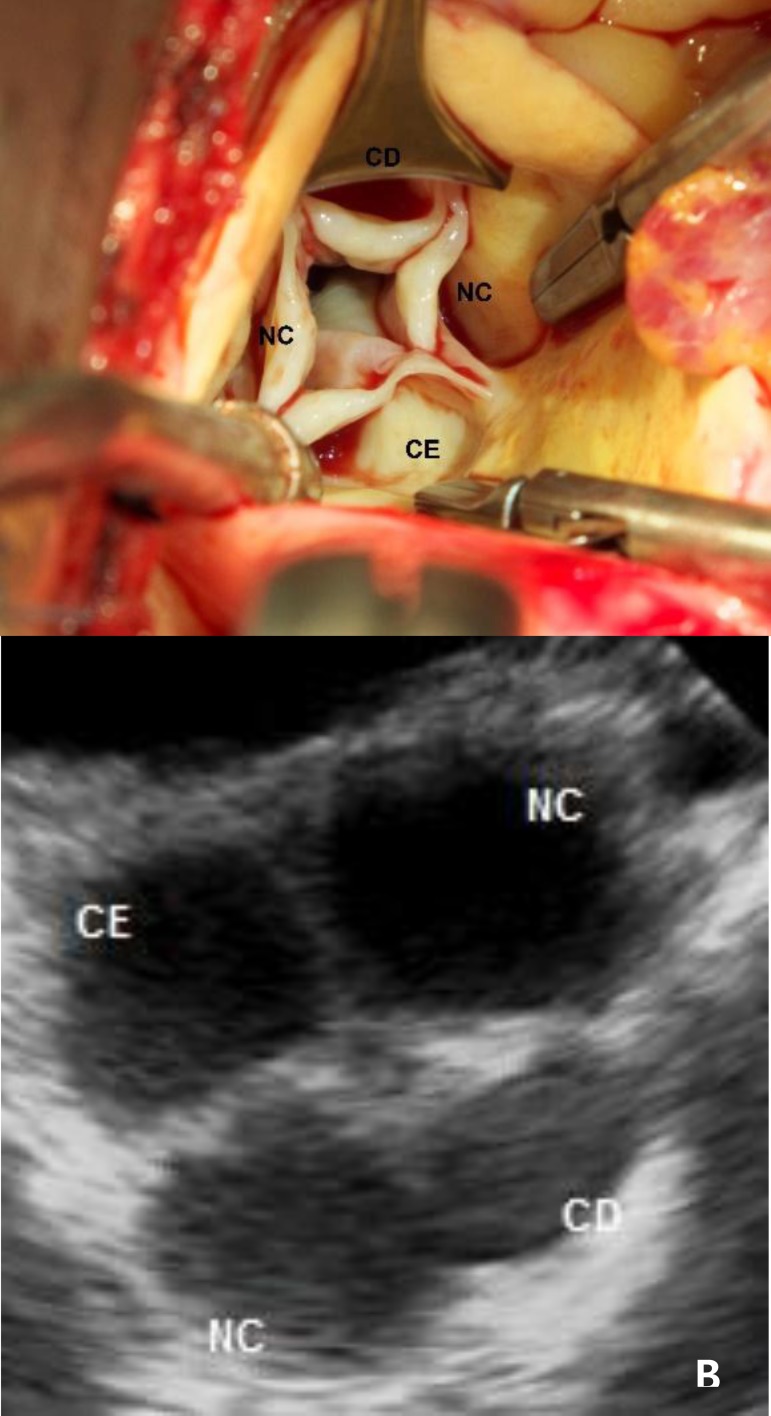
(A). Intraoperative view of Type-B Hurwitz and Roberts QAV. (B)Transesophageal echocardiogram showing the four cusps. CE=left coronary cusp; CD=right coronary cusp; NC=non coronary cusp.

A 51 year-old female patient with a progressive worsening of symptoms of fatigue and dyspnea in the previous 12 months was admitted to our institution for an elective surgery. At the time, she had a marked limitation of physical activity. The physical examination demonstrated a diastolic murmur +3/+6 at the left middle sternal border. Transthoracic echocardiogram revealed a severe insufficient aortic valve with LVEF preserved (71%). Signs of malcoaptation were noticed between the cusps, although an accurate anatomical characterization was not possible due to limitation of the acoustic window in the transverse plane. The intraoperative transesophageal echocardiogram revealed a quadricuspid aortic valve (Figure 3B). In the surgical field, the aortic valve was composed by four retracted cusps and it was classified as a Type B in the Hurwitz and Roberts classification. There were three equal cusps and one smaller cusp (Figure 3A). The patient underwent a minimally invasive aortic valve surgery through superior mini-sternotomy with an "Inverted L" incision in the fourth right intercostal space. The extracorporeal circulation was placed in both right femoral artery and vein. The aortic valve was replaced by a Braile^®^ 23mm Bovine Pericardium Bioprosthesis. The procedure was successful, and the patient had an excellent in-hospital postoperative recovery and remains asymptomatic after 12 months of follow-up.

## DISCUSSION

QAV is a rare congenital heart disease with an incidence of 0,008% to 0,033% in autopsy series and 0,013-0,043% for patients undergoing transthoracic echocardiographic examinations^[2]^.

The mechanisms of QAV development remain unclear. The main hypothesis is the abnormal septation of embryological arterial trunk. Abnormal cusp formation occurs either to aberrant fusion of the aorticopulmonary septum or to abnormal proliferations in the common trunk^[1]^. It is usually an isolated anomaly, but other congenital heart defects can be present in 18-32% of the patients and coronary artery and coronary ostium anomalies are the most frequent associated disorders^[3]^.

The most widely used classification is the one by Hurwitz and Roberts^[1]^, which divides 7 alphabetical subtypes based on the cusps size: type A - 4 identical leaflets, type B - 3 identical leaflets and 1 smaller leaflet, type C - 2 larger identical leaflets and 2 smaller identical leaflets, type D - 1 larger leaflet, 2 intermediate leaflets and 1 smaller leaflet, type E - 3 identical leaflets and 1 larger leaflet, type F - 2 larger identical leaflets and 2 smaller non-identical leaflets, type G - 4 non-identical leaflets. According to these authors, about 87% of cases presented the types A, B or C.

On echocardiogram, the quadricuspid aortic valve is identified by its characteristic “X” shape during diastole (different from the “Y” of the standard trivalvular aortic valve) and its rectangular shape during systole. It tends to evolve to failure over decades due to the asymmetry in the distribution of transvalvar flow and inequality in coaptation of the leaflets^[4]^. For this reason, some cases of mixed valve dysfunction have been described, but the finding of pure valvular stenosis is very rare. Besides, a small supernumerary cusp can be a predictive risk factor of infective endocarditis^[3]^.

The surgical treatment is indicated for symptomatic patients, left ventricular dysfunction and left ventricular remodeling in the presence of important dysfunctional aortic valve. The decision about valvular repair or replacement depends on the valvular characteristics and the surgeons experience. Aortic valve repair is performed in about 25% of cases in which the tricuspidization accounts for 80% of the repair procedures, especially in the Type B valves^[5,6]^.

In our cases, the aortic valve replacement was the surgical choice due to the severe structural disorder on the native valves. Patients with QAV may be followed-up to early detect the beginning or the worsening of clinical conditions and prompt surgical treatment assure good mid-to-long term results.

**Table:** 

Authors' roles & responsibilities	
ETV	Substantial contributions to the conception or design of the work; or the acquisi-tion, analysis, or interpretation of data for the work; final approval of the version to be published
CMAB	Substantial contributions to the conception or design of the work; or the acquisi-tion, analysis, or interpretation of data for the work; final approval of the version to be published
SPS	Substantial contributions to the conception or design of the work; or the acquisi-tion, analysis, or interpretation of data for the work; final approval of the version to be published
PP	Drafting the work or revising it critically for important intellectual content; final approval of the version to be published
FJ	Final approval of the version to be published
